# Modeling the Relationships Between Metacognitive Beliefs, Attention Control and Symptoms in Children With and Without Anxiety Disorders: A Test of the S-REF Model

**DOI:** 10.3389/fpsyg.2019.01205

**Published:** 2019-06-07

**Authors:** Marie Louise Reinholdt-Dunne, Andreas Blicher, Henrik Nordahl, Nicoline Normann, Barbara Hoff Esbjørn, Adrian Wells

**Affiliations:** ^1^Department of Psychology, University of Copenhagen, Copenhagen, Denmark; ^2^Department of Psychology, Norwegian University of Science and Technology, Trondheim, Norway; ^3^School of Psychological Sciences, University of Manchester and Greater Manchester Mental Health NHS Trust, Manchester, United Kingdom

**Keywords:** anxiety disorders, childhood anxiety, metacognition, attention control, prevention, psychological treatment

## Abstract

In the metacognitive model, attentional control and metacognitive beliefs are key transdiagnostic mechanisms contributing to psychological disorder. The aim of the current study was to investigate the relative contribution of these mechanisms to symptoms of anxiety and depression in children with anxiety disorders and in non-clinical controls. In a cross-sectional design, 351 children (169 children diagnosed with a primary anxiety disorder and 182 community children) between 7 and 14 years of age completed self-report measures of symptoms, attention control and metacognitive beliefs. Clinically anxious children reported significantly higher levels of anxiety, lower levels of attention control and higher levels of maladaptive metacognitive beliefs than controls. Across groups, lower attention control and higher levels of maladaptive metacognitive beliefs were associated with stronger symptoms, and metacognitions were negatively associated with attention control. Domains of attention control and metacognitions explained unique variance in symptoms when these were entered in the same model within groups, and an interaction effect between metacognitions and attention control was found in the community group that explained additional variance in symptoms. In conclusion, the findings are consistent with predictions of the metacognitive model; metacognitive beliefs and individual differences in self-report attention control both contributed to psychological dysfunction in children and metacognitive beliefs appeared to be the strongest factor.

## Introduction

Anxiety disorders are the most common psychological problems in children and adolescents with prevalence estimates ranging from 3–20% (Costello et al., 2005; Cartwright-Hatton et al., 2006). They are associated with considerable developmental, psychosocial and psychopathological complications (Beesdo et al., 2009). For example, anxiety has a negative impact on school functioning (Mychailyszyn et al., 2010) and is a major risk factor for developing comorbid disorders such as depression (Wittchen et al., 2003). Moreover, childhood anxiety disorders predict psychopathology in adolescence and adulthood (Bittner et al., 2007; Copeland et al., 2009), and are associated with substantial functional impairment in later life (Copeland et al., 2014). Hence, identifying factors underlying anxiety disorders and vulnerability to developing them may inform the development of effective prevention- and treatment interventions that could benefit individuals and society.

Cognitive theories of anxiety implicate biases in information processing in the development and maintenance of anxiety (e.g., Williams et al., 1988). Such biases can be observed in the content of interpretations of experience where the sense of danger and threat predominate (Beck et al., 1985). They are also evident at the level of attentional processes, where anxiety and depression are associated with biased attention for negative emotion-related stimuli (Bar-Haim et al., 2007; Cisler and Koster, 2010). A major challenge is to identify the factors that give rise to bias in psychological disorders. Early models viewed bias as the result of automatic or reflexive processes, but this has been questioned. For example, Wells and Matthews (1994) proposed specific multiple influences on bias including metacognitive beliefs and the individual’s goals and strategies for self-regulation of which volitional attention control is a major component. Attention control has been conceptualized as the ability to control attention in inhibiting a dominant response in favor of a less accessible, subdominant response that may be more functional (Rothbart and Bates, 1998; Derryberry and Reed, 2002). Thus, attention control is viewed as a self-regulatory capacity, and it has been shown to moderate the association between attentional bias for threat and anxiety in adults (e.g., Derryberry and Reed, 2002; Bardeen and Orcutt, 2011) and in children (e.g., Lonigan and Vasey, 2009; Susa et al., 2012). Consequently, individual differences in attention control could contribute to resilience or vulnerability to emotional distress (e.g., Lonigan et al., 2004; Muris et al., 2004, 2007, 2008; Susa et al., 2012).

The role of such influences of attention and their link with emotional vulnerability has been captured in detail in the Self-Regulatory Executive Function (S-REF) model (Wells and Matthews, 1994, 1996), which is the basis of the metacognitive model of emotional disorder and treatment (Wells, 2009). In this model, psychological dysfunction is associated with a style of thinking called the cognitive attentional syndrome (CAS). The hallmark of the CAS is perseverative thinking consisting of worry/rumination, threat monitoring and maladaptive coping strategies. The activation and persistence of the CAS is dependent on underlying metacognitive knowledge (i.e., beliefs about cognition). Metacognitive knowledge refers to the information that individuals hold about their own cognition and internal states (e.g., “my worrying thoughts are uncontrollable”) and about coping strategies (e.g., “worrying helps me to get things sorted out in my mind”) and is implicated across mental disorders (Wells, 2009; Sun et al., 2017). Such metacognitions also contribute to psychological vulnerability when the presence of a mental disorder has been accounted for Nordahl and Wells (2017).

Within the S-REF model, attention control is considered a general resource that facilitates cognitive regulation and the ability to disengage from conceptual processing and perseverative self-focused attention (i.e., the CAS). Whilst this ability is separate from but related to the effects of metacognitive knowledge (Wells and Matthews, 1994) individual differences in attention control (i.e., executive functions) could affect the individual’s ability to disengage from the CAS. Attentional control is likely to be comprised at least in part of knowledge or beliefs about attention and studies separating the effects of attention performance (skills) and beliefs about attention in psychological disorder are lacking.

In the metacognitive model in particular, beliefs about poor attention control are of interest as they are likely to be part of a broader dysfunctional metacognitive knowledge base hypothesized to underlie psychological disorder. Furthermore, different dimensions of metacognition may interact and increase the risk or severity of psychological disorder symptoms. In particular, high levels of perceived attention control could help to ameliorate the negative effects of beliefs about the dangerousness of thoughts on anxiety. In contrast, low levels of perceived attention control might enhance the negative effect of metacognitions about the uncontrollability and danger of worrying.

In adults, studies have found support for an association between greater maladaptive metacognitive beliefs and lower perceived attention control (Spada et al., 2010; O’Carroll and Fisher, 2013; Spada and Roarty, 2015; Fernie et al., 2016), and both perceived attention control and metacognitive beliefs have been found to explain unique variance in performance test anxiety (O’Carroll and Fisher, 2013), state anxiety in students before end-of-year examinations (Spada et al., 2010), and decisional procrastination (Fernie et al., 2016). Moreover, Fergus et al. (2012) found that the relationships between activation of the CAS and symptoms became increasingly stronger as self-reported attention control decreased, indicating that activation of the CAS is associated with especially deleterious effects for individuals with low attention control.

In sum, attention control (beliefs) and other metacognitive beliefs may be central to understanding psychological disorder and vulnerability. However, research on the relationship between attention control and metacognitive beliefs and their individual or combined contribution to symptoms is scarce, and to the authors’ knowledge has not been investigated in children. The aim of the current study was therefore to investigate the relative contribution of attention control and metacognitive beliefs in children with anxiety disorders and in non-clinical controls. We set out to examine differences between community controls and clinical patients and to explore the unique and interactive effects of metacognitive beliefs and attention control within each group. Our hypotheses were as follows; (1) the clinical group will report greater severity of symptoms, lower attention control and higher levels of maladaptive metacognitive beliefs than the control group; (2) attention control will be negatively associated with symptoms; (3) metacognitive beliefs will be positively associated with symptoms; (4) metacognitive beliefs will be negatively associated with attention control; (5) attention control and metacognitive beliefs will account for unique variance in symptoms; (6) there should be an interaction between metacognitive beliefs and attention control that contributes to symptoms. Because we cannot predict based on theory whether the interaction occurs in non-patients and/or patients we tested the model in the clinical and non-clinical groups separately.

## Materials and Methods

### Participants and Procedure

A child community sample was recruited by sending invitation letters to 1601 families with children aged 8 to 12 years of age living within the catchment area of Center for Anxiety, Department of Psychology, University of Copenhagen. The sample was randomly selected by the Danish Central Office of Civil Registration, and the invitation letter specified that only typically developing children could participate. Families that wished to participate completed a questionnaire booklet online at home, prior to entering the clinic. When entering the clinic, mothers completed the parent version of the Anxiety Disorders Interview Schedule (ADIS; Silverman and Albano, 1996). The interview showed that all participating children were free of psychiatric disorders.

In the clinical sample, families referred their preadolescent children to the clinic, although they had often been recommended to contact the clinic by other professionals, e.g., psychiatrists and school psychologists. Consequently, preadolescents in aged between 7 and 14 that had a primary anxiety disorder, either generalized anxiety disorder, separation anxiety disorder, specific phobia, or social phobia were eligible as participants for the study if they also had an IQ above 70, and one parent native speaker of Danish. The children were assessed with the ADIS (Silverman and Albano, 1996). A combined diagnosis was derived from child and parent ratings, and showed that 110 (65.1%) of the children fulfilled the diagnostic criteria for generalized anxiety disorder, 33 (19.5%) for separation anxiety disorder, 14 (8.3%) for social phobia, and 12 (7.1) for specific phobia. The majority of the children (141; 83.4%) had comorbid anxiety disorders. Sixteen children (9.5%) also had comorbid mood disorder (Dysthymia or Major depressive disorder). A total of 351 children participated in this study, 182 community children (100 girls; 54.9%) between 7 and 12 years of age (*M* = 10.00, *SD* = 1.40) and 169 children diagnosed with a primary anxiety disorder (89 girls; 52.7%) between 7 and 14 years of age (*M* = 9.93, *SD* = 1.83) were included. Comparison of the community and clinical groups using Chi square and independent *t*-tests (on categorical and continuous variables, respectively) showed no significant group differences in gender or age distribution between the two groups.

### Ethics Statement

Ethical approval for the study was obtained from the Institutional Review Board at the Department of Psychology, University of Copenhagen. The study complies with ethical standards in the 1964 Helsinki declaration and its later amendments regarding assessment and treatment for children enrolled in psychological research studies. Written informed consent to participate was obtained from all parents of participating youth, and assent was obtained from the youth.

### Measures

The Revised Child Anxiety and Depression Scale – Child version (RCADS, Chorpita et al., 2000) is a 47-item self-report questionnaire measuring child anxiety and depression symptoms. RCADS consists of six subscales: Major depression, social phobia, panic disorder, separation anxiety, generalized anxiety, and obsessive-compulsive disorder. The major depression subscale consists of ten items, the social phobia and the panic disorder subscales consist of nine items, the separation anxiety subscale consists of seven items, and the generalized anxiety and the obsessive-compulsive disorder subscales consist of six items. A total score can be computed by summing the subscales. Validation of the Danish version of RCADS has shown satisfactory psychometric properties (Esbjørn et al., 2012). In the current study, internal consistency was excellent in both the community group (α = 0.94) and the clinical group (α = 0.93).

Attentional Control Scale for Children (ACS-C; Derryberry and Reed, 2002) is a 20-item self-report questionnaire measuring subjective attentional control. ACS-C consists of three subscales: Attention focusing, attention shifting, and flexible control of thought. The attention focusing subscale consists of nine items, the attention shifting subscale consists of six items, and the flexible control of thought subscale consists of five items. Items have to be scored on a 4-point scale with 1 = never, 2 = sometimes, 3 = often, and 4 = always. After recoding inversely formulated items, a total score can be computed by summing the subscales. The ACS-C has shown acceptable psychometric properties (e.g., Muris et al., 2004, 2007, 2008). In this study internal consistency was satisfactory in the clinical group (α = 0.74) and slightly below satisfactory level in the community group (α = 0.57).

Metacognitions Questionnaire for Children (MCQ-C_30_, Esbjørn et al., 2013) is a 30-item self-report questionnaire measuring metacognitive beliefs and processes in children and is a simplified version of the original adult scale (Wells and Cartwright-Hatton, 2004). MCQ-C_30_ consists of five subscales: Positive beliefs about worry, negative beliefs about uncontrollability and danger of worry, cognitive confidence, need for control, and cognitive self-consciousness. All the subscales consist of six items. Items are scored on a 4-point scale with 1 = do not agree, 2 = agree slightly, 3 = agree moderately, and 4 = agree very much. A total score can be computed by summing the subscales. The Danish version of the questionnaire has shown satisfactory psychometric properties (Esbjørn et al., 2013). In the present study internal consistency was satisfactory in both the community group (α = 0.89) and the clinical group (α = 0.86).

### Overview of Data Analyses

Independent samples *t*-tests were used to compare the community and the clinical group on the RCADS, and on the subscales of the ACS-C and the MCQ-C_30_. Then we ran bivariate correlational analyses to investigate the relationship between these variables.

To explore if there was any interaction effect between attention control and metacognitive beliefs on symptoms, we used structural equation modeling (Bentler, 1995). MCQ-C_30_ total score, ACS-C total score, and the interaction between these two were used as observed variables, while symptoms (RCADS) was used as a latent variable consisting of all the RCADS subscales. Evaluation of the path coefficient from the interaction variable to the latent construct symptoms was of particular interest, as a significant path coefficient would indicate that moderation occurred.

Hierarchical linear regression analyses were run in each group to test the relative contribution of the attention control domains and metacognitive belief domains. Moreover, if the SEM analysis revealed a moderation effect, we planned to add this interaction variable to the regression models as a means to evaluate its relative contribution over domains of attention control and metacognitive beliefs. RCADS was used as the dependent variable throughout. Gender and age was controlled in the first step. In the second step, we entered the ACS-C subscales, and the MCQ-C_30_ subscales were entered in step 3. If the SEM analyses revealed moderation, we planned to enter the interaction variable on the fourth step to test whether the interaction between metacognitive beliefs and attention control explained additional variance in the final equation when unique effects of attention control and metacognitive beliefs were controlled.

## Results

### Group Comparisons

We found significant differences between the groups in symptom severity (RCADS total score), in all three domains of attention control, and in all domains of metacognitive beliefs except for judgments of cognitive confidence; the clinical group scored significantly higher on symptoms and metacognitive beliefs, and significantly lower on attention control compared to the community group. Descriptive statistics and group comparisons are presented in Table 1.

**Table 1:** Group comparisons between the community- and the clinical group on age, symptom severity (RCADS), attentional control (ACS-C), and metacognitive beliefs (MCQ-C_30_); mean score, standard deviation and *t*-value.

	Community group (*n* = 182)		Clinical group (*n* = 169)		
	Mean	Std.	Mean	Std.	*t*-Value
Age	10.00	1.40	9.93	1.83	0.681
RCADS	21.34	14.34	48.03	20.69	13.945**
ACS-C-total	57.34	5.80	49.27	7.98	–10.770**
ACS-C-focus	25.95	3.40	22.50	4.73	–7.812**
ACS-C-shifting	17.77	2.57	16.08	2.78	–5.928**
ACS-C-flexible	13.62	2.62	10.70	2.73	–10.240**
MCQ-C_30_-total	42.88	9.46	55.67	10.95	11.731**
MCQ-C_30_-pos	6.92	1.43	8.15	2.34	5.857**
MCQ-C_30_-neg	8.50	2.85	14.00	3.82	15.205**
MCQ-C_30_-cc	9.07	2.69	9.62	3.16	1.748
MCQ-C_30_-nc	8.37	2.30	10.67	2.90	8.191**
MCQ-C_30_-csc	10.00	3.19	13.23	3.76	8.644**


*RCADS, revised child anxiety and depression scale – child version; ACS-C, attention control scale for children; focus, attention focusing; shifting, attention shifting; flexible, flexible control of thought; MCQ-C_*30*_, metacognitions questionnaire for children; pos, positive metacognitive beliefs; neg, negative metacognitive beliefs; cc, cognitive confidence; nc, need for control; csc, cognitive self-consciousness. ^∗∗^*p* < 0.01.*

### Correlational Analyses

In both groups, there was a significant association between RCADS and ACS-C focusing and shifting, indicating that lower levels of attention control in these two domains are associated with higher levels of symptoms, while there was no association between RCADS and the ACS-C flexible subscale in any of the groups. RCADS was significantly associated with all domains of metacognitive beliefs in the community group, and with all but positive metacognitive beliefs in the clinical group, indicating that higher levels of symptoms are associated with higher maladaptive metacognitive beliefs. Moreover, lower levels of attention control were associated with higher levels of maladaptive metacognitive beliefs; the ACS-C focusing subscale was significantly and negatively associated with all domains of metacognitive beliefs in the community group, while it was significantly negatively associated with all metacognitive belief domains except positive metacognitive beliefs in the clinical group. The ACS-C shifting subscales was significantly and negatively associated with negative metacognitive beliefs and judgments of cognitive confidence in both groups, and with need for control in the clinical group, but not with positive metacognitive beliefs or cognitive self-consciousness in any of the groups or need for control in the community group. The ACS-C subscale flexible control of thought was significantly and negatively correlated with negative metacognitive beliefs and cognitive confidence in the clinical group, but was not associated with metacognitions in the community group. The bivariate correlations are presented in Table 2.

**Table 2:** Bivariate correlations between age, RCADS total score, ACS-C subscales, and MCQ-C_30_ subscales in the community- and the clinical group.

	Community group (*n* = 182)									Clinical group (*n* = 169)								
	2	3	4	5	6	7	8	9	10	2	3	4	5	6	7	8	9	10
1. Age	–0.01	–0.03	0.15*	–0.06	0.10	0.08	0.11	0.09	0.10	–0.01	0.13	0.03	0.16*	–0.10	0.03	0.10	0.09	0.07
2. RCADS		–0.50**	–0.34**	0.10	0.36**	0.68**	0.49**	0.53**	0.57**		–0.49**	–0.30**	–0.14	0.09	0.62**	0.35**	0.55**	0.43**
3. ACS-C-focus			0.43**	0.07	–0.20**	–0.37**	–0.37**	–0.26**	–0.31**			0.46**	0.34**	–0.07	–0.46**	–0.42**	–0.39**	–0.25**
4. ACS-C-shifting				–0.01	–0.07	–0.22**	–0.26**	–0.12	0.12				0.35**	0.01	–0.16*	–0.40**	–0.21**	–0.06
5. ACS-C-flexible					–0.03	–0.01	0.11	–0.01	–09					0.04	–0.18*	–0.19*	–0.13	–0.07
6. MCQ-C_30_-pos						0.29**	0.20**	0.44**	0.40**						0.05	–0.00	0.19*	0.12
7. MCQ-C_30_-neg							0.38**	0.61**	0.63**							0.29**	0.56**	0.54**
8. MCQ-C_30_-cc								0.44**	0.32**								0.45**	0.27**
9. MCQ-C_30_-nc									0.68**									0.57**
10. MCQ-C_30_-csc																		


*RCADS, revised child anxiety and depression scale – child version; ACS-C, attention control scale for children; focus, attention focusing; shifting, attention shifting; flexible, flexible control of thought; MCQ-C_*30*_, metacognitions questionnaire for children; pos, positive metacognitive beliefs; neg, negative metacognitive beliefs; cc, cognitive confidence; nc, need for control; csc, cognitive self-consciousness. ^∗^*p* < 0.05, ^∗∗^*p* < 0.01.*

### Structural Equation Modeling

Structural equation modeling (Bentler, 1995) was used to investigate if there was an interaction effect between attention control and metacognitive beliefs in predicting distress in each group. The total score from the ACS-C and MCQ-C_30_ together with their interaction (ACS-C total score × MCQ-C_30_ total score) were treated as observed variables, and symptoms were treated as a latent variable consisting of each of the RCADS subscales. The path coefficients were of particular interest, and if the path from the interaction variable to the dependent variable had no predictive value, it was deleted to evaluate a second model without the interaction. Evaluation of overall model fit was conducted according to Hu and Bentler (1999), where the Comparative Fit Index (CFI) and the Tucker-Lewis Index (TLI) should be close to or more than 0.95, the standardized root mean square residual (SRMR) should be less than 0.08, and the root mean square error of approximation (RMSEA) should be less than 0.06, to represent good model fit.

**FIGURE 1:**
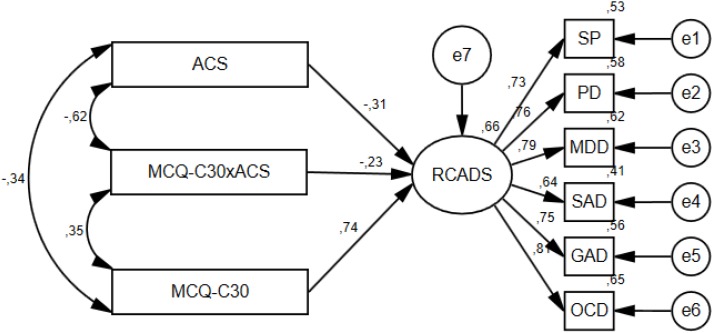
Structure and standardized estimates of the best fitting model in the community group.

In the community group, the data fitted the model reasonably well when the interaction variable was included as the CFI and SRMR were within recommendations, the TLI was borderline of its recommended value, while the RMSEA was above recommended value; χ^2^(24) = 52.315, *p* = 0.001, CFI = 0.964, TLI = 0.947, RMSEA = 0.081, SRMR = 0.0367. All the standardized regression weights in the model were significant at the 0.001 level, which indicated that there was an additional interaction effect between attention control and metacognitive beliefs in the community sample. In this model, the squared multiple correlation for symptoms (RCADS) was 0.66, indicating that 66% of the variance in symptoms was accounted for by the predictors. We also evaluated the model fit in the community sample without the interaction term, and this model also fitted well, showing slightly better fit indices than the first model: χ^2^(19) = 41.233, *p* = 0.002, CFI = 0.968, TLI = 0.952, RMSEA = 0.080, SRMR = 0.0365. However, a chi square difference test showed that the model with the interaction variable was significantly better than the model without the interaction term: Δχ^2^ = 11.082, Δdf = 5, (*p* < 0.05). The model with the interaction variable in the community sample is presented in Figure 1.

**FIGURE 2:**
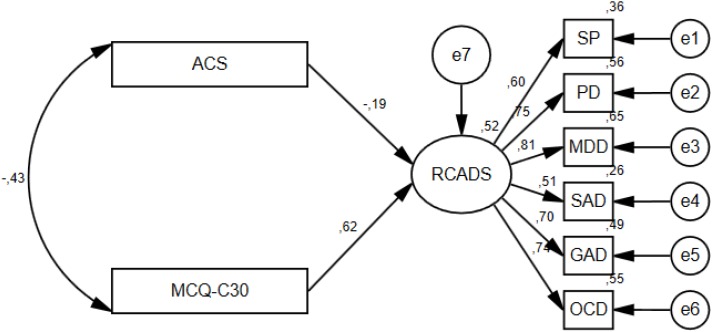
Structure and standardized estimates of the best fitting model in the clinical group.

In the clinical sample, the path from the interaction variable (attention control x metacognitive beliefs) to symptoms was non-significant, indicating that there was no additional contribution of the interaction effect. The interaction variable was therefore deleted before evaluating the model fit. All standardized regression weights in this second model were significant at 0.01 level and the squared multiple correlation for symptoms (RCADS) was 0.52, indicating that 52% of the variance in symptoms was accounted for by the predictors. Still, the model did not provide an optimal fit to the data in the clinical group; χ^2^(19) = 48.644, *p* < 0.000, CFI = 0.942, TLI = 0.914, RMSEA = 0.096, SRMR = 0.0479. The model without the interaction variable in the clinical group is presented in Figure 2.

### Hierarchical Linear Regression Analyses

In the community group, gender and age was not a significant predictor of symptoms in any of the steps in the regression model. On the second step, all domains of attention control made unique contributions to symptoms and together they explained an additional 27% of the variance. In the second step, metacognitive beliefs explained 34% of the variance in symptoms over and above the attention control domains. Need for control was non-significant as a predictor, but all other domains of metacognitive beliefs were significant predictors, and negative metacognitive beliefs explained most variance. Adding metacognitive beliefs to the model led the shifting subscale of the ACS-C to be non-significant as a predictor, while the two other attention control subscales remained significant indicating that they accounted for unique variance in symptoms. Building on the SEM-analysis, the interaction effect was entered in the model in the fourth step and explained an additional 1 % of the variance. In this final step of the equation, attention control focusing and shifting, negative metacognitive beliefs, cognitive confidence, cognitive self-consciousness and positive metacognitive beliefs together with the interaction variable remained significant predictors and explained unique variance in symptoms.

In the clinical group, age was non-significant as a predictor of symptoms, while gender was significant as a predictor in the first step, showing that female gender was associated with higher levels of symptoms. In the second step, the ACS-C focusing subscale was significant as a predictor of symptoms, while the two other ACS-C subscales were not. In sum, attention control accounted for an additional 24 % of the variance in this step. Furthermore, gender remained a significant predictor of symptoms in the second step. In the third step, metacognitive beliefs explained an additional of 22 % of the variance in symptoms over and above age/gender and attention control. Of the MCQ-C_30_ subscales, negative metacognitive beliefs and need for control were significant individual predictors. After adding metacognitive beliefs to the model, the ACS-C focusing subscales remained significant as a predictor, while gender became non-significant. The regressions are presented in Table 3.

**Table 3:** Hierarchical regression analysis in the community- and clinical group separately, with RCADS total score as the dependent, gender/age and subscales from the ACS-C and MCQ-C_30_ as predictors.

	Community group (*n* = 182)					Clinical group (*n* = 169)				
Criterion variable	Step	*R*^2^	*R*^2^ change	Predictor	β	Step	*R*^2^	*R*^2^ change	Predictor	β
RCADS	1	0.01	0.01			1	0.04	0.04*		
				Gender	0.12				Gender	0.19*
				Age	–0.01				Age	–0.02
	2	0.28	0.27**			2	0.28	0.24**		
				Gender	0.06				Gender	0.16*
				Age	0.01				Age	0.04
				ACS-C-focus	–0.43**				ACS-C-focus	–0.46**
				ACS-C-shifting	–0.15*				ACS-C-shifting	–0.10
				ACS-C-flexible	0.13*				ACS-C-flexible	0.06
	3	0.63	0.34**			3	0.50	0.22**		
				Gender	0.04				Gender	0.08
				Age	–0.06				Age	–0.03
				ACS-C-focus	–0.18**				ACS-C-focus	–0.18*
				ACS-C-shifting	–0.09				ACS-C-shifting	–0.12
				ACS-C-flexible	0.12*				ACS-C-flexible	0.08
				MCQ-C_30_-pos	0.12*				MCQ-C_30_-pos	0.02
				MCQ-C_30_-neg	0.39**				MCQ-C_30_-neg	0.35**
				MCQ-C_30_-cc	0.17**				MCQ-C_30_-cc	0.03
				MCQ-C_30_-nc	–0.01				MCQ-C_30_-nc	0.21**
				MCQ-C_30_-csc	0.18*				MCQ-C_30_-csc	0.06
	4	0.64	0.01*							
				Gender	0.04					
				Age	–0.06					
				ACS-C-focus	–0.22**					
				ACS-C-shifting	–0.13*					
				ACS-C-flexible	0.07					
				MCQ-C_30_-pos	0.12*					
				MCQ-C_30_-neg	0.37**					
				MCQ-C_30_-cc	0.17**					
				MCQ-C_30_-nc	0.01					
				MCQ-C_30_-csc	0.21**					
				MCQxACS	–0.14*					


*RCADS, revised child anxiety and depression scale – child version; ACS-C, attention control scale for children; focus, attention focusing; shifting, attention shifting; flexible, flexible control of thought; MCQ-C_*30*_, metacognitions questionnaire for children; pos, positive metacognitive beliefs; neg, negative metacognitive beliefs; cc, cognitive confidence; nc, need for control; csc, cognitive self-consciousness; β, standardized beta coefficients. ^∗^*p* < 0.05, ^∗∗^*p* < 0.01.*

**FIGURE 3:**
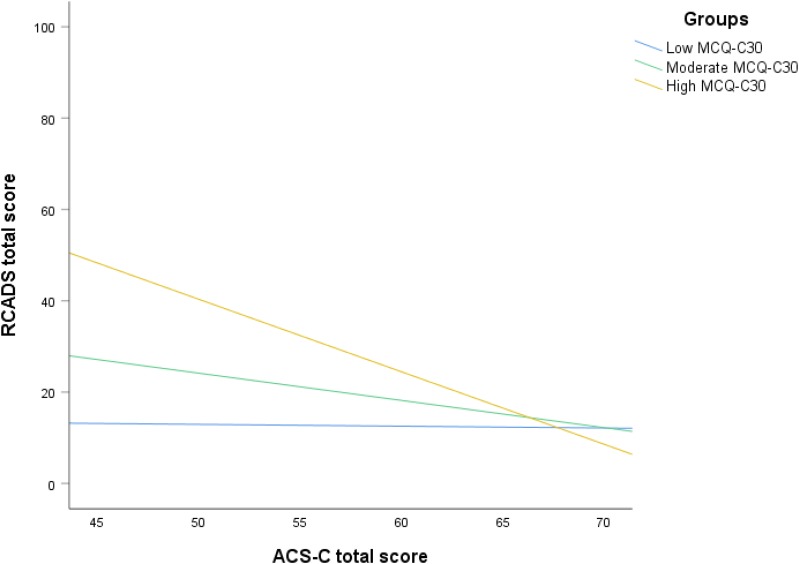
Scatter plot showing the effect of attention control on symptoms at different levels of metacognition.

**FIGURE 4:**
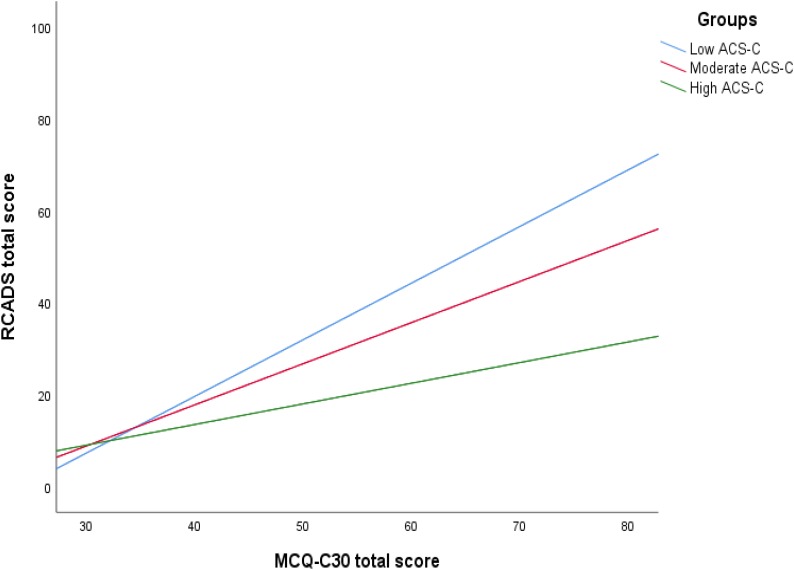
Scatter plot showing the effect of metacognition on symptoms at different levels of attention control.

To further explore the interaction effect in the community group, we examined two scatter plots with symptoms (RCADS total score) represented along the Y-axis. In the first scatter plot, attention control (ACS-C total score) were represented along the X-axis. The participants were separated in three group based on their total MCQ-C_30_ score; group 1 consisted of the lowest scoring one-third of the sample; group 2 consisted of the one third of the individuals that had a moderate score; group 3 consisted of the one third of the individuals with the highest score. In the second scatter plot, metacognitive beliefs (MCQ-C_30_ total score) were represented along the X-axis, and the sample was divided in low, moderate and high attention control groups following the same principle as outlined above. The scatter plots are presented in Figure 3, 4.

Inspection of the plots shows that as dysfunctional metacognitions increase from moderate to high the negative relationship between attention control and symptoms becomes stronger. There is no effect at low levels of metacognitions. Conversely, at higher levels of attention control, the positive relationship between metacognitions and symptoms becomes weaker but there is no effect at low levels of attention control.

## Discussion

Metacognitive beliefs and attention control are two influences on cognitive regulation that have been implicated in the metacognitive model of psychological disorders. This model predicts differences between these factors in clinical and non-patient individuals, for example that higher endorsements of maladaptive metacognitive beliefs and lower attentional control abilities should be found in clinical compared to non-clinical samples. It also predicts that these factors should be positively associated with symptoms of anxiety and depression in both groups, and that they may interact to moderate the strength of association each of these factors has with anxiety symptoms.

As predicted, the clinical child group showed elevated scores on dysfunctional metacognitive beliefs and lower scores on attention control compared to community controls. Negative metacognitive beliefs about worry differentiated the most between the groups among all predictors, while confidence in memory did not differentiate between the groups. Among the attention control dimensions, the flexible control of thought subscale differentiated the most between groups.

Within each group, we found the expected positive relationship between symptom severity and metacognitive beliefs, with the strongest association with negative metacognitive beliefs about worry. There was no association with positive beliefs in the clinical group, but all other relationships between metacognitive belief domains and symptoms were significant in both groups and showed relationships of moderate or moderate to low strength. The expected negative relationship between symptoms and attention control was evident as attention focusing and shifting showed a moderate to low association with symptoms, while the relationship with the flexible control of thoughts subscale was non-significant in both groups.

With the exception of positive metacognitive beliefs in the clinical sample, attention focusing was negatively associated with all domains of metacognitive beliefs in both groups. The same relationship was observed between attention shifting, negative metacognitive beliefs and cognitive confidence in both groups, and also between attention shifting and need for control in the clinical group, indicating that maladaptive metacognitive knowledge is related to lower (perceived) ability to control attention.

On testing for interaction effects, the interaction between the total score on the MCQ-C_30_ and the ACS-C, was found in the community- but not the clinical sample. This is an interesting finding because it suggests that a multiplicative effect of metacognitions and attention control on symptoms might be most relevant to sub-clinical anxiety and depression symptoms (at least in children). This raises an intriguing but speculative possibility, but one that is nonetheless congruent with the metacognitive model; that strongly held maladaptive metacognitive beliefs can neutralize the emotional benefit conferred by strong attention control beliefs, or conversely that strong attention control can remediate the negative effects of strongly held maladaptive metacognitive beliefs. But these findings point to a possible mechanistic or process-based difference between clinical and non-clinical samples. The interaction was not observed in the clinical group, one explanation might be that the deleterious effects attributed to high metacognitions is not moderated or offset by attentional control in those who have clinical disorder because their dysfunctional metacognitions are so much greater or these individuals use less effective mental regulation strategies. Such effects would be consistent with the S-REF model where attention control and flexibility is considered a general purpose processing resource that is compromised by high dysfunctional metacognitions and strategy selection (i.e., using extended negative thinking to deal with stress) (Wells and Matthews, 1994).

When exploring the relative contribution from the individual ACS-C and MCQ-C_30_ subscales in the community sample, attention focusing and shifting, positive metacognitive beliefs, negative metacognitive beliefs, low cognitive confidence, cognitive self-consciousness and the additional interaction effect between metacognitions and attention control explained unique variance in symptoms. In the clinical sample, attention focusing, negative metacognitive beliefs and beliefs about the need to control thoughts were the only significant independent contributors to symptoms, indicating that greater negative beliefs about the uncontrollability and danger of thoughts, need to control thoughts, and lower levels of attention focusing contribute individually to greater symptoms in clinically anxious children. While positive metacognitive beliefs are suggested to be an important disposition to anxiety disorder specific in metacognitive models of anxiety (Wells, 2009), we found that there was no independent effect of positive metacognitive beliefs on anxiety in the clinical group. One explanation could be that the effects of positive beliefs was masked by the substantial contribution from negative metacognitive beliefs and need for control, that are a more proximal contributor to disorder. Moreover, it could be that different domains of metacognitions may serve as maintenance factors (i.e., negative metacognitive beliefs and need for control) and as causal factors constituting vulnerability (i.e., positive metacognitive beliefs) as reported by others (e.g., Nordahl et al., 2019), but this possibility cannot be tested given the cross-sectional data-set in the present study.

In sum, our findings suggest that the metacognitive model might offer a useful framework to conceptualize psychopathology and psychological vulnerability in children with the implication that metacognitive therapy techniques for the prevention- and treatment of disorder could be applicable. Metacognitive therapy (Wells, 2009) interventions aim to modify maladaptive metacognitive knowledge and strengthen flexible control over attention and they should be investigated in this group. While cognitive-behavioral therapy (CBT) is an effective treatment for anxiety in children (e.g., Ewing et al., 2015; James et al., 2015), regulatory processes (i.e., metacognition) and executive function aspects are in large overlooked in these models and treatments, which may account for the fact that a substantial number of patients are non-responders (James et al., 2005). For example, Reinholdt-Dunne et al. (2015) found that attention control did not significantly change in anxious children following CBT. Furthermore, the effect size of anxiety prevention programs for children has been reported as small (e.g., Fisak et al., 2011), indicating a need for further therapeutic developments. Applications of metacognitive therapy and techniques for children have begun and show promising results (Simons et al., 2006; Esbjørn et al., 2015; Murray et al., 2016, 2018; Simons and Kursawe, 2019). However, more studies are needed before any firm conclusions on its effect can be drawn.

The present study has several limitations that should be acknowledged. First, a cross-sectional design was used, and therefore no causal inferences can be made. Second, the clinical sample in this study predominantly consisted of children with primary GAD, and our study should therefore be replicated in a wider clinical context. Furthermore, the clinical sample was a convenience sample of preadolescents referred for treatment, which resulted in a heterogeneous sample in terms of both primary diagnoses and age. While this is a limitation in some respect, our study has external validity as the sample consisted of patients referred to a clinic setting. Third, self-report symptom assessment is a limitation of the study. Fourth, an important question concerns the measurement of attention control. In a recent study, the ACS was largely unrelated to behavioral performance measures of attention control (Williams et al., 2017), indicating that the ACS may represent subjective judgments of attention (metacognitive knowledge) rather than actual cognitive ability. However, metacognitive beliefs have been associated with objective shifting ability after controlling for symptoms and general cognitive function (Kraft et al., 2017) and improved neuropsychological functioning has been observed following MCT for depression (Groves et al., 2015) in adults, suggesting that there is a link between metacognitions (including beliefs about attention) and some aspects of objective executive functioning. Further research should utilize longitudinal and experimental designs with objective measures of attention control to better address the relation and direction of relations among metacognitive beliefs, objective attention control and psychopathology symptoms. In addition, testing the contribution of attentional control and metacognitive knowledge to symptoms in more specific clinical groups of children may further enhance our understanding. Further research should take account of potential age differences when exploring the influence of metacognitive knowledge and executive functions on psychological disorder and vulnerability in children.

## Conclusion

In conclusion, metacognitive beliefs and attention control appear to contribute to emotion disorder symptoms in both clinical and non-clinical children samples. This suggests that prevention strategies and treatment interventions should aim to modify maladaptive metacognitive knowledge and enhance judgments of attention control as recommended in metacognitive therapy. But the nature of the relationship between objective attention performance, beliefs about attention control and disorder symptoms remains to be differentiated.

## Ethics Statement

Ethical approval for the study was obtained from the Institutional Review Board at the Department of Psychology, University of Copenhagen. The study complies with ethical standards in Denmark regarding assessment and treatment for children enrolled in psychological research studies. Written informed consent to participate was obtained from all parents of participating youth, and assent was obtained from the youth.

## Author Contributions

All authors were part of the design of the study. MR-D, AB, NN, and BE were part of data collection and writing the manuscript. HN and AW were part of analyzing the data and writing the manuscript.

## Conflict of Interest Statement

The authors declare that the research was conducted in the absence of any commercial or financial relationships that could be construed as a potential conflict of interest.
